# Exploring perceptions of OCR technology adoption for paper document archiving in Saudi Arabia: an exploratory study

**DOI:** 10.3389/fdata.2026.1916400

**Published:** 2026-10-29

**Authors:** Alia Mohammed AlSulaimi

**Affiliations:** Department of Information Technology, College of Computer and Information Sciences, Imam Mohammad Ibn Saud Islamic University (IMSIU), Riyadh, Saudi Arabia

**Keywords:** digital transformation, document archiving, optical character recognition (OCR), Saudi Arabia, technology acceptance model (TAM), vision 2030

## Abstract

The ongoing digital transformation agenda introduced under Saudi Vision 2030 has encouraged organizations across the Kingdom to modernize document management practices and reduce dependence on paper-based records. Within this context, Optical Character Recognition (OCR) technology has become an important enabler of document digitisation by supporting the conversion of printed records into searchable digital content. Despite its potential, the implementation of OCR in Saudi organizations continues to face a number of organizational, technical, and linguistic challenges, particularly when processing Arabic-language documents and integrating OCR into existing archival workflows. This study investigated the adoption of OCR technology for document archiving in Saudi Arabian organizations through the theoretical perspective of the Technology Acceptance Model (TAM). A descriptive cross-sectional design was employed using a structured questionnaire that included both closed-ended items and optional open-ended responses. Data were collected from two participant groups comprising domain experts (*n* = 8) and general technology users (*n* = 15) representing public and private sector organizations. The findings indicate that participants generally viewed OCR technology positively, particularly with regard to its perceived usefulness. Document retrieval efficiency received unanimous agreement from both participant groups, identifying faster access to archived information as the most widely recognized benefit of OCR adoption. Simultaneously, respondents identified several factors that continue to hinder wider implementation. These included limited access to specialized OCR training and concerns about digitizing fragile or historical paper records. Challenges associated with Arabic script are considered in the manuscript as contextual issues identified in the existing literature rather than as direct empirical findings of the present survey. Participants also reported that although OCR improves document retrieval and day-to-day workflow efficiency, its integration into organizational processes still depends on adequate technical infrastructure, workforce preparedness, and effective implementation planning. The findings offer practical insights that may support future organizational digitisation initiatives aligned with the objectives of Saudi Vision 2030. Overall, the study provides exploratory descriptive evidence on participants' perceptions of OCR adoption, implementation challenges, and perceived organizational impact within Saudi Arabian document-archiving contexts. The study contributes to the limited empirical literature examining the organizational and user-centered dimensions of OCR adoption in Arabic-language archival environments.

## 1 Introduction

### 1.1 Background and technological evolution of OCR

The widespread adoption of digital technologies has fundamentally reshaped the way organizations create, manage, store, and access information. As administrative processes continue to shift toward digital environments, effective records management has become a strategic organizational function rather than a routine administrative task (Asem et al., 2024; Benmakhlouf and Chouaou, 2024). At the same time, the continuous growth of organizational data, together with increasing expectations for regulatory compliance, efficient information retrieval, and improved operational performance, has encouraged organizations to adopt document digitisation technologies capable of transforming paper-based archives into searchable digital repositories (Gowrishankar and Praveen, 2023; Moudgil et al., 2022).

Among the technologies supporting this transformation, Optical Character Recognition (OCR) has emerged as one of the most widely implemented solutions for document digitisation and archival management. OCR converts printed or handwritten text contained in scanned documents into machine-readable digital content, enabling documents to be searched, indexed, and processed electronically (Gowrishankar and Praveen, 2023; Liu, 2026). Modern OCR systems integrate techniques from image processing, pattern recognition, artificial intelligence, machine learning, and deep learning to improve recognition performance under a wide range of operating conditions (Gowrishankar and Praveen, 2023). As noted by He (2020), these capabilities have enabled OCR applications to expand beyond document digitisation into areas such as office automation, intelligent information processing, machine translation, and monitoring systems. Consequently, OCR has evolved from relatively simple rule-based recognition software into intelligent systems capable of processing multilingual documents across diverse application domains.

The technological development of OCR has accelerated considerably during the past decade. Earlier OCR systems relied largely on template matching and heuristic recognition methods, which often performed poorly when processing degraded documents, irregular fonts, or noisy images (Moudgil et al., 2022). Subsequent advances introduced statistical sequence-modeling approaches, including Hidden Markov Models (HMMs) and Conditional Random Fields (CRFs), resulting in noticeable improvements in recognition accuracy (Moudgil et al., 2022). More recently, deep learning models based on Convolutional Neural Networks (CNNs), Recurrent Neural Networks (RNNs), and Long Short-Term Memory (LSTM) networks trained with Connectionist Temporal Classification (CTC) have substantially reduced the dependence on explicit character segmentation while improving recognition performance (Mosbah et al., 2024; Abudaqqa, 2025). Transformer architectures and vision-language models have further extended OCR capabilities, particularly for multilingual collections and specialized document types such as Arabic and historical manuscripts (Liu, 2026; Hegghammer, 2022).

Despite these technological advances, OCR performance continues to vary according to document quality, language characteristics, and operational conditions. Comparative evaluations involving Tesseract, Amazon Textract, and Google Document AI indicate that cloud-based OCR services generally achieve higher recognition accuracy when processing degraded or noisy documents (Hegghammer, 2022). Nevertheless, open-source OCR solutions remain widely adopted because of their flexibility and lower implementation costs. For instance, Jayoma et al. (2020) demonstrated that integrating PyTesseract with Django and MySQL can effectively support automated document classification, indexing, and archival workflows within organizational environments.

Processing Arabic documents remains considerably more challenging than processing Latin-based scripts. Arabic writing is inherently cursive, context-sensitive, and characterized by multiple character forms, ligatures, and optional diacritical marks, all of which increase the complexity of segmentation and character recognition (Najam and Faizullah, 2023; Faizullah et al., 2023). Although recent deep learning techniques have significantly improved Arabic OCR performance, important challenges remain, particularly in handwritten text recognition, domain adaptation, model robustness, and the availability of annotated datasets suitable for administrative and archival documents (Najam and Faizullah, 2023; Kasem et al., 2026). In the present study, these Arabic-script characteristics are treated as contextual factors identified in the existing literature rather than as experimentally evaluated OCR performance measures.

The literature has consistently reported several organizational benefits associated with OCR implementation. Converting scanned documents into searchable digital records can substantially reduce the time and effort required to retrieve organizational information (Raj et al., 2023; Hegghammer, 2022). Previous studies have also associated OCR adoption with improvements in workflow efficiency, document indexing, classification accuracy, regulatory compliance, and information accessibility (Benmakhlouf and Chouaou, 2024). Furthermore, OCR contributes to the long-term preservation of historical and administrative records by facilitating the creation of sustainable digital archives (Hegghammer, 2022; Zhang, 2024).

Although these benefits are well documented, successful implementation remains influenced by both technical and organizational factors. Recognition accuracy may decline when OCR systems process low-resolution images, deteriorated paper documents, handwritten annotations, irregular layouts, or multilingual records commonly encountered in archival collections (Sandnes, 2022). Likewise, post-recognition correction often depends on language-specific processing techniques, particularly for Arabic administrative documents (Mosbah et al., 2024). Beyond technical performance, organizational readiness also plays an important role. Factors such as infrastructure availability, employees' digital competencies, training opportunities, resistance to organizational change, and existing document management practices can all influence the successful adoption of OCR technologies (Alharbi, 2019).

### 1.2 Study context, theoretical framework, and research objectives

Saudi Arabia provides a particularly relevant context for investigating the organizational adoption of Optical Character Recognition (OCR) technology. Over the past decade, the country has pursued an ambitious digital transformation agenda aimed at modernizing governmental services and organizational operations. The launch of Saudi Vision 2030 accelerated national initiatives related to e-government, administrative automation, digital service delivery, and information governance, creating strong momentum for the digitisation of organizational records and business processes (Asem et al., 2024; ع. ب. ع. ا. الزهراني ع. ب. ع. ا. الشيحة, 2025). As organizations continue to replace paper-based procedures with digital workflows, interest in OCR has increased because of its potential to improve document accessibility, support archival modernization, and facilitate information retrieval across institutional repositories (Abudaqqa, 2025). Despite these developments, several practical challenges continue to influence OCR implementation, particularly those associated with Arabic-language document processing, organizational readiness, and workforce capabilities (Abudaqqa, 2025).

To examine these issues, this study adopts the Technology Acceptance Model (TAM) as its primary theoretical framework. Since its introduction by Davis (1989), TAM has been widely applied to explain users' acceptance of information technologies through two central constructs: perceived Usefulness (PU), which reflects the extent to which a technology is believed to improve job performance, and Perceived Ease of Use (PEOU), which represents users' perceptions of the effort required to use that technology effectively. These constructs provide an appropriate foundation for examining how organizational members evaluate OCR systems within document archiving environments.

Although OCR has become an increasingly common component of enterprise document management systems, empirical evidence describing users' perceptions of OCR adoption within Saudi organizations remains limited. Existing research has largely concentrated on improving recognition algorithms and benchmarking OCR accuracy, while comparatively less attention has been given to understanding how technological capabilities interact with organizational conditions and user experiences in everyday archival practice. Consequently, there remains a need for empirical evidence that captures these organizational perspectives within the Saudi context.

Accordingly, this study investigates the current adoption of OCR technology for paper-based document archiving across public and private sector organizations in Saudi Arabia. Guided by the conceptual principles of TAM (Davis, 1989), the study examines participants' perceptions of the benefits of OCR, the challenges associated with its implementation, its perceived organizational impact, and the factors influencing its integration into existing archival workflows. Rather than testing causal relationships or validating structural models, the research adopts a descriptive perspective intended to provide an evidence-based understanding of current OCR adoption practices.

The study is guided by the following overarching research question:

- How do participants in Saudi Arabian organizations perceive the adoption of OCR technology for paper-document archiving in terms of current adoption, perceived usefulness and ease of integration, implementation challenges, and perceived organizational impact?
- The study objectives are:
- RO1: To examine the current adoption of OCR technology and participants' perceptions of its usefulness and ease of integration within paper-document archiving workflows.
- RO2: To identify the principal organizational, technical, and operational challenges perceived to affect OCR implementation and integration within Saudi Arabian archival environments.
- RO3: To describe the perceived organizational impact of OCR adoption and interpret the findings using the Technology Acceptance Model as a conceptual and interpretive framework.

The conceptual framework underpinning this study views OCR adoption as the outcome of interactions between technological capabilities and organizational conditions. Consistent with TAM, perceptions of usefulness and ease of use are interpreted alongside contextual factors identified in the information systems literature, including the complexity of Arabic script, infrastructure readiness, and operational requirements within organizational archiving environments. To investigate these dimensions, the study employs a descriptive cross-sectional research design supported by frequency distributions, percentage analysis, weighted mean scores, and reliability assessment. These analytical procedures are consistent with the descriptive objectives of the study and provide an appropriate basis for interpreting participants' responses.

This study makes contributions at several levels. From a practical perspective, it provides baseline evidence that may assist organizations in planning document digitisation initiatives and supporting the broader objectives of Saudi Vision 2030 (Asem et al., 2024; ع. ب. ع. ا. الزهراني ع. ب. ع. ا. الشيحة, 2025). From an academic perspective, it addresses a relatively underexplored area concerning the organizational and user-centered dimensions of OCR adoption in Saudi Arabian document-archiving environments, while applying TAM as an interpretive framework within a non-Western context. From an applied perspective, the study provides contextual evidence on how OCR is perceived and integrated within organizational archiving practices, with particular attention to perceived usefulness, ease of integration, implementation challenges, and perceived organizational impact.

The remainder of this paper is organized as follows. Section 2 reviews the literature on OCR technologies, document digitisation, and organizational adoption. Section 3 describes the research methodology, including the sampling strategy, research instrument, data collection procedures, and analytical methods. Section 4 presents the empirical findings, while Section 5 discusses the results in relation to the existing literature and the study's theoretical framework. Finally, Section 6 summarizes the main conclusions and outlines directions for future research.

## 2 Review of related literature

This section critically examines the body of scholarship related to the adoption, implementation, and organizational implications of OCR technologies within digital archiving environments. Rather than presenting descriptive summaries of prior studies, the review adopts an analytical synthesis approach that integrates technical, organizational, and user-centered perspectives in order to establish the conceptual foundation of the present study. The reviewed literature is organized into six interrelated themes: (1) OCR Systems, Tools, and Comparative Evaluation; (2) OCR in Document Archiving and Records Management; (3) OCR Applications in High-Stake Information Environments; (4) Arabic OCR: Advances, Challenges, and Limitations; (5) User Experience and Technology Acceptance in Digital Systems; and (6) Digital Transformation and OCR Adoption in the Saudi Arabian Context. Collectively, these thematic areas clarify the current state of knowledge while identifying the empirical and theoretical gaps that motivate the present research.

### 2.1 OCR systems, tools, and comparative evaluation

Recent advances in deep learning, transformer architectures, and intelligent document processing have contributed substantially to the development of OCR technologies (Gowrishankar and Praveen, 2023; Moudgil et al., 2022; Liu, 2026). Modern OCR solutions increasingly combine computer vision, sequence modeling, and natural language processing techniques to improve text recognition and contextual interpretation across a wide range of document types (Gowrishankar and Praveen, 2023; Liu, 2026). Nevertheless, OCR performance continues to be influenced by several factors, including document quality, preprocessing procedures, script characteristics, and deployment conditions (Moudgil et al., 2022; Hegghammer, 2022; Faizullah et al., 2023).

A number of benchmarking studies have compared the performance of commercial and open-source OCR platforms, particularly in multilingual and archival settings. Hegghammer (2022) evaluated Tesseract, Amazon Textract, and Google Document AI using more than 18,000 Arabic and English document images subjected to different levels of degradation. The findings showed that cloud-based platforms generally achieved higher recognition accuracy than traditional open-source solutions when processing noisy documents. However, lower recognition rates were observed for Arabic texts across all evaluated systems, suggesting that improvements in OCR performance remain uneven across languages (Hegghammer, 2022). This observation is particularly relevant to the current study, given the prevalence of Arabic-language records in Saudi organizational archives.

The literature also indicates that OCR effectiveness is shaped by more than the recognition engine alone. Studies have shown that preprocessing quality, image enhancement, noise removal, and output formatting can substantially affect the usability of OCR-generated content within document management workflows (Moudgil et al., 2022; Hegghammer, 2022). Similarly, Gowrishankar (Gowrishankar and Praveen, 2023) and Liu (2026) noted that recent gains in recognition accuracy are increasingly associated with multimodal learning approaches and context-aware architectures rather than isolated character-recognition techniques. At the same time, challenges related to computational requirements, domain adaptation, scalability, and model reliability continue to be reported (Gowrishankar and Praveen, 2023; Liu, 2026).

Overall, contemporary OCR research has gradually moved beyond evaluating isolated recognition accuracy. Current studies increasingly examine OCR as part of a broader document-processing ecosystem in which preprocessing, workflow integration, contextual interpretation, and multilingual support collectively influence operational performance. This perspective is particularly applicable to organizational archiving environments where document quality, formatting standards, and language requirements vary considerably.

### 2.2 OCR in document archiving and records management

Research on OCR-enabled archiving consistently highlights the role of OCR as a component of broader records management and information-governance initiatives rather than a standalone recognition technology (Benmakhlouf and Chouaou, 2024; Hegghammer, 2022; Jayoma et al., 2020). Across different institutional settings, OCR adoption has been associated with faster document retrieval, improved indexing practices, reduced manual processing effort, and greater accessibility to archived information (Benmakhlouf and Chouaou, 2024; Jayoma et al., 2020). Such benefits are particularly important in organizations that maintain extensive paper-based records and depend on efficient information retrieval.

Jayoma et al. (2020) developed an OCR-based archiving and indexing platform using PyTesseract to support governmental records management. Their findings demonstrated that searchable digital repositories can improve retrieval and indexing processes while reducing reliance on manual document handling. The authors also emphasized that recognition accuracy remained dependent on image quality and preprocessing consistency, indicating that successful implementation requires adjustments to existing workflows rather than software deployment alone (Jayoma et al., 2020).

Related findings were reported by Sandnes (2022), who examined OCR-based accessibility solutions. The study showed that recognition performance declined when documents contained complex layouts, degraded images, or substantial graphical content. Certain document categories remained difficult to process even under advanced OCR implementations, highlighting the influence of document structure and archival conditions on system effectiveness (Sandnes, 2022).

Organizational integration has also emerged as an important theme within the literature. Benmakhlouf and Chouaou (2024) reported that interoperability between OCR systems, archival repositories, and institutional databases contributed to improved workflow continuity and operational efficiency. Their findings suggest that the effectiveness of OCR adoption is closely linked to infrastructure readiness, system compatibility, and process integration capabilities rather than recognition performance alone (Benmakhlouf and Chouaou, 2024).

Evidence from studies involving complex and low-resource scripts further supports this view (Najam and Faizullah, 2023; Faizullah et al., 2023; Kasem et al., 2026). Although deep-learning approaches have achieved notable improvements in recognition accuracy, researchers continue to report difficulties associated with document degradation, segmentation instability, and low-resolution imagery (Faizullah et al., 2023; Kasem et al., 2026). These challenges resemble many of the conditions found in archival collections and reinforce the need to view OCR-enabled archiving as a socio-technical process shaped by both technological and organizational factors.

### 2.3 OCR applications in high-stake information environments

Healthcare is among the most frequently studied domains for OCR implementation because of its dependence on extensive documentation, stringent accuracy requirements, and ongoing digital transformation efforts (Kushwaha et al., 2025; Batra et al., 2024; Gifu, 2022). Although healthcare workflows differ from archival management systems, studies in this field provide useful insights into the practical value, implementation requirements, and operational challenges associated with OCR technologies.

Research in healthcare settings indicates that recognition performance is influenced not only by the OCR engine but also by the quality of image preparation. Batra et al. (2024) evaluated several OCR engines using different image enhancement techniques and reported that preprocessing operations such as binarisation, filtering, brightness adjustment, and image sharpening improved recognition accuracy during medical record digitisation. Their findings suggest that successful OCR deployment requires attention to configuration, optimisation, and workflow design in addition to software selection (Batra et al., 2024).

Other studies have highlighted the importance of post-processing and information structuring. Bouh et al. (2024) demonstrated that reorganizing OCR-generated text into coherent reading sequences improved the readability and usability of digitized medical records. Similarly, Kushwaha et al. (2025) examined AI-assisted OCR systems for medical label interpretation and argued that contemporary OCR platforms increasingly support information extraction and contextual analysis rather than simple text transcription. This trend reflects the growing integration of OCR with artificial intelligence techniques designed to enhance semantic understanding and decision support (Kushwaha et al., 2025).

Gifu (2022) also explored the role of AI-enhanced OCR in healthcare environments and noted its potential for processing handwritten clinical information and automating transcription activities. The study further observed that recognition errors may influence operational reliability and information quality, thereby emphasizing the need for effective validation and quality-control mechanisms (Gifu, 2022).

Taken together, these studies indicate that perceptions of OCR usefulness are influenced by a combination of recognition accuracy, workload reduction, information accessibility, and workflow efficiency. Similar considerations are relevant within organizational archiving environments, where large document volumes and ongoing digitalisation initiatives create comparable operational requirements.

### 2.4 Arabic OCR: advances, challenges, and limitations

Arabic OCR continues to present distinctive technical challenges due to the linguistic and structural characteristics of the Arabic script (Najam and Faizullah, 2023; Faizullah et al., 2023; Kasem et al., 2026). Unlike many Latin-based writing systems, Arabic writing is cursive and context dependent, with character shapes changing according to their position within a word. These characteristics increase the complexity of segmentation, feature extraction, and recognition processes, particularly when dealing with handwritten or degraded archival materials (Najam and Faizullah, 2023; Faizullah et al., 2023).

Recent developments in deep learning have contributed to noticeable improvements in Arabic OCR performance. Najam and Faizullah (2023) reported that CNN-LSTM-CTC architectures achieved favorable results in handwritten Arabic text recognition by effectively modeling contextual relationships within text sequences. Likewise, advances in convolutional and bidirectional sequence-learning approaches have contributed to higher recognition accuracy in Arabic document processing tasks (Mosbah et al., 2024).

Despite these developments, several challenges remain unresolved. Previous studies continue to report difficulties associated with segmentation errors, font variation, diacritical ambiguity, image degradation, and limited domain adaptation for administrative and archival documents (Najam and Faizullah, 2023; Faizullah et al., 2023; Kasem et al., 2026). Kasem et al. (2026) further identified the limited availability of large annotated datasets for governmental and archival records as an important constraint affecting the development of robust Arabic OCR solutions.

Post-recognition correction represents another area where challenges persist. Although language-model-based correction methods have improved OCR output quality in several languages, equivalent advances in Arabic post-processing remain comparatively limited (Najam and Faizullah, 2023). As a result, OCR systems may exhibit higher error propagation rates when applied to real-world Arabic documents than those reported under controlled evaluation conditions.

These concerns are supported by comparative benchmarking evidence. Hegghammer (2022) found that Arabic recognition accuracy declined more noticeably than English recognition accuracy when document quality deteriorated. Considering that many organizational archives contain aging documents, handwritten annotations, and inconsistent formatting, these limitations represent practical deployment challenges that extend beyond algorithmic performance alone. Successful implementation therefore depends on the combined influence of technical capability, language-specific adaptation, preprocessing quality, and organizational workflow requirements.

### 2.5 User experience and technology acceptance in digital systems

The effectiveness of OCR implementation is influenced not only by technical performance but also by how users perceive and interact with the technology. For this reason, the TAM provides a useful framework for examining OCR adoption within organizational environments (Davis, 1989; Badda, 2025; McCord, 2007).

Originally proposed by Davis (1989), TAM explains technology acceptance through two principal constructs: PU and PEOU. Perceived Usefulness reflects the extent to which users believe that a system enhances job performance, whereas Perceived Ease of Use relates to the effort associated with learning and operating the technology (Davis, 1989). Subsequent developments of TAM expanded the framework to incorporate additional organizational and contextual influences relevant to contemporary digital environments (Badda, 2025; McCord, 2007).

Evidence from technology-adoption research suggests that positive usability perceptions are frequently associated with greater acceptance and sustained system use. Studies examining technology-supported environments (Jebeile and Reeve, 2007; Hornbæk and Hertzum, 2017) found that users generally report higher satisfaction when systems align with established work practices and minimize operational complexity. Similar observations have been reported in studies involving non-technical users, where intuitive interfaces and simplified interactions contributed to favorable adoption experiences (Hornbæk and Hertzum, 2017).

Research across the information systems field also indicates that user acceptance is shaped by factors such as interface clarity, perceived efficiency, reliability, and workflow compatibility (Davis, 1989; (Badda, 2025; McCord, 2007; Jebeile and Reeve, 2007; Hornbæk and Hertzum, 2017). These findings suggest that technology adoption is influenced by both functional performance and the broader user experience surrounding system operation.

Within the context of OCR, this perspective implies that adoption outcomes are likely to reflect more than recognition accuracy alone. Perceived workload reduction, ease of integration, reliability, and organizational support may all influence how users evaluate OCR systems. Although TAM has been widely applied across different technological contexts, relatively few studies have explored OCR adoption perceptions within Arabic-language archival environments, particularly in Saudi Arabian organizations undergoing digital transformation initiatives.

### 2.6 Digital transformation and OCR adoption in the Saudi Arabian context

The broader digital transformation literature provides an important context for understanding OCR adoption within Saudi Arabia (Alharbi, 2019; Asem et al., 2024; ع. ب. ع. ا. الزهراني ع. ب. ع. ا. الشيحة, 2025; Abudaqqa, 2025). Existing studies indicate that the country has made considerable progress in digital governance and technology-driven service delivery, particularly following the introduction of Vision 2030 and its associated modernization initiatives (Asem et al., 2024; Alharbi, 2019; ع. ب. ع. ا. الزهراني ع. ب. ع. ا. الشيحة, 2025).

Alharbi (2019) noted that organizational transformation efforts in Saudi Arabia continue to be influenced by factors such as workforce readiness, variations in digital literacy, and the persistence of legacy administrative structures. While national initiatives have accelerated the adoption of digital technologies across both public and private sectors, the transition of internal document-management and records-processing functions has progressed at different rates across organizations (Asem et al., 2024; Abudaqqa, 2025).

Studies examining Saudi Arabia's digital transformation landscape further highlight the role of government-supported digital platforms in expanding electronic services and improving administrative efficiency (ع. ب. ع. ا. الزهراني ع. ب. ع. ا. الشيحة, 2025; Abudaqqa, 2025). At the same time, researchers continue to identify challenges related to system integration, organizational change management, employee preparedness, and process redesign (Alharbi, 2019; Asem et al., 2024). These issues are particularly relevant to OCR implementation because document archiving frequently involves long-established paper-based procedures and institutional practices that may require substantial adaptation during digital transition processes.

Another recurring theme within the literature concerns the relationship between technology adoption and organizational context. Existing evidence suggests that the outcomes of digital transformation initiatives are shaped not only by technological capability but also by leadership commitment, organizational culture, infrastructure readiness, and employee engagement (Alharbi, 2019; Asem et al., 2024). This perspective is directly applicable to OCR adoption, where successful implementation depends on the interaction between system performance, organizational preparedness, and user acceptance.

Taken together, the Saudi Arabian literature suggests that OCR implementation should be viewed within the broader context of organizational digital transformation. Understanding how institutions perceive the benefits, challenges, and operational impact of OCR may therefore contribute to a more comprehensive understanding of technology adoption within contemporary Saudi workplaces.

### 2.7 Synthesis and identification of research gaps

The reviewed literature demonstrates that OCR technologies have advanced considerably and now form an integral component of modern document-management and information-processing systems. Previous studies have documented the potential of OCR to improve document accessibility, support workflow automation, enhance retrieval processes, and facilitate the preservation of organizational records across a variety of operational settings. At the same time, the literature identifies several challenges that continue to influence the effectiveness of OCR implementation in practice.

From a technical perspective, Arabic OCR remains less mature than many OCR solutions developed for Latin-based scripts. Studies consistently report difficulties related to document degradation, segmentation errors, font variation, diacritical ambiguity, and the limited availability of specialized datasets for governmental and archival materials (Hegghammer, 2022; Najam and Faizullah, 2023), (Faizullah et al., 2023), (Kasem et al., 2026). Although recent deep-learning approaches have improved recognition performance, several of these challenges remain evident in real-world deployment environments.

At the organizational level, the literature highlights the importance of infrastructure readiness, workflow compatibility, staff capabilities, and institutional support in shaping technology-adoption outcomes (Liu, 2026; Asem et al., 2024; Abudaqqa, 2025). While these factors have been examined within broader digital transformation research, relatively limited attention has been given to their influence on OCR implementation in organizational archiving environments, particularly within Saudi Arabian institutions.

A further gap emerges from the theoretical literature. Although the Technology Acceptance Model has been widely applied to investigate user perceptions of digital technologies, its use in OCR-related studies remains comparatively limited. Much of the existing OCR literature continues to focus on algorithmic performance, benchmarking experiments, and recognition accuracy, with less emphasis placed on users' perceptions, organizational experiences, and operational adoption conditions (Gowrishankar and Praveen, 2023; Moudgil et al., 2022; Liu, 2026; Kasem et al., 2026; Zhou, 2026).

The applied literature also reveals a contextual limitation. Many OCR studies have been conducted in laboratory environments, specialized technical domains, or consumer-oriented applications. Consequently, less is known about how OCR technologies are perceived and utilized within professional records-management and document-archiving contexts, particularly where multilingual records, organizational procedures, and regulatory requirements interact simultaneously.

These observations suggest a need for research that examines OCR adoption from a broader organizational perspective. In the Saudi Arabian context, where digital transformation initiatives continue to reshape administrative practices, there remains a limited body of empirical evidence describing how organizations perceive the benefits, challenges, ease of integration, and operational impact of OCR technologies. Addressing this gap may contribute to a more contextualized understanding of OCR implementation within Arabic-language archival environments.

The specific gap addressed by the present study is therefore not the technical recognition performance of Arabic OCR, but the limited empirical understanding of how OCR is perceived, integrated, and associated with perceived organizational outcomes within Saudi Arabian document-archiving environments. In particular, relatively limited attention has been given to the perspectives of organizational users and domain experts regarding perceived usefulness, ease of integration, implementation challenges, and perceived organizational impact. The present study addresses this contextual and user-centered gap through an exploratory descriptive survey rather than through experimental evaluation of Arabic OCR recognition accuracy.

Accordingly, the present study adopts a descriptive approach to examine OCR adoption across Saudi Arabian organizations. Drawing upon the Technology Acceptance Model as an interpretive framework, the study explores perceived usefulness, ease of integration, implementation challenges, and organizational impact through descriptive statistical analysis (Field, 2018) and qualitative feedback. By combining technological, organizational, and user-centered perspectives, the study seeks to provide an evidence-based overview of OCR adoption within contemporary archival environments.

## 3 Methodology

This section outlines the methodological framework adopted for the study, including the research design, sampling strategy, survey instrument, data collection procedures, and analytical methods used to examine the adoption of OCR technology in document archiving environments within Saudi Arabian organizations. The study was designed to explore participants' perceptions of OCR-related benefits, implementation challenges, ease of integration, and perceived organizational impact from the perspectives of both domain experts and general technology users.

### 3.1 Research design

The study employed a descriptive cross-sectional design based on a structured questionnaire that also included an optional open-ended response section. This approach was considered appropriate because the research focuses on describing participants' perceptions and experiences of OCR technology within a specific organizational setting rather than testing causal relationships. Cross-sectional survey designs are widely used in information systems research to examine perceptions and attitudes among different participant groups at a particular point in time (Creswell and Creswell, 2017; Saunders et al., 2019).

Data were collected from both OCR specialists and general users during the same period, providing an overview of current OCR adoption experiences across different Saudi Arabian organizations. The quantitative component relies primarily on descriptive statistical techniques, including frequencies, percentages, and weighted mean scores, which are commonly applied in exploratory and technology-adoption research where the objective is to describe response patterns and identify broad trends rather than establish causal effects (Badda, 2025; Creswell and Creswell, 2017; Saunders et al., 2019). A summary of the methodological workflow and data-analysis process is presented in Figure 1.

**Figure 1:**
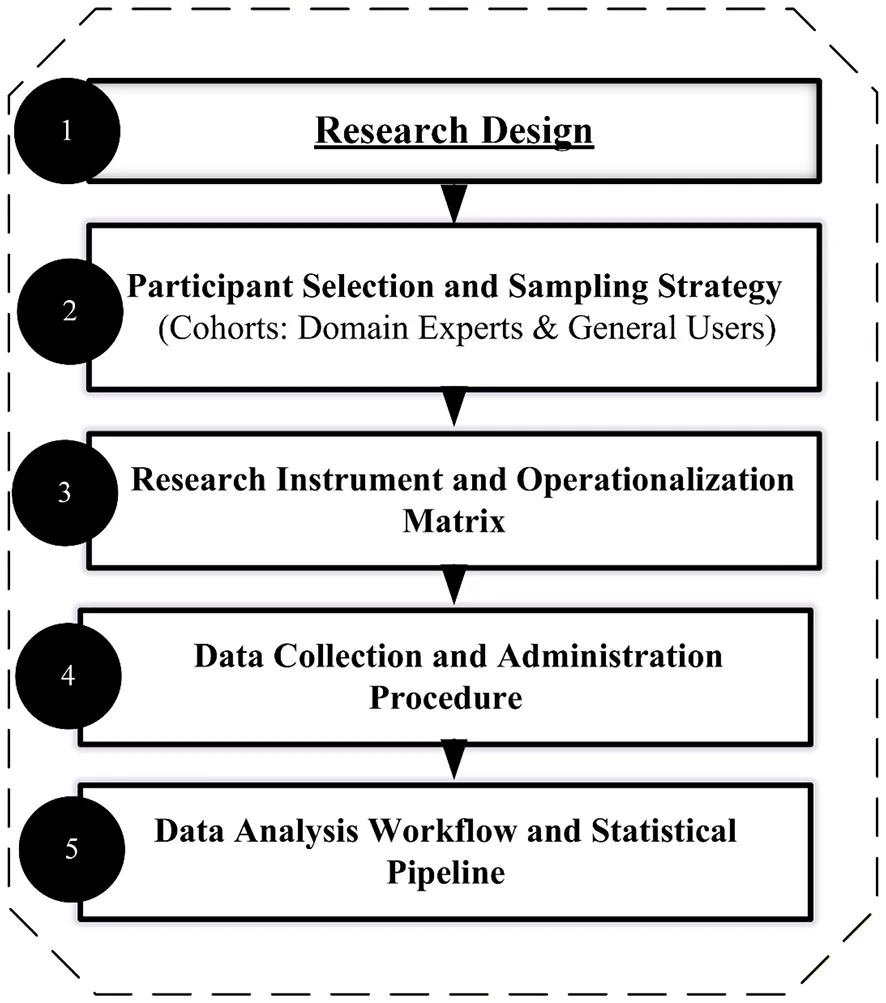
Methodological workflow and data analysis pipeline.

The selection of a descriptive quantitative approach was guided by three considerations.

**First**, the target population of OCR practitioners and specialists in Saudi Arabia remains relatively limited, which restricts the feasibility of applying more complex inferential techniques that typically require substantially larger samples. A descriptive approach therefore provides a more appropriate basis for interpreting the collected data.

**Second**, the study aims to document and contextualize participants' perceptions regarding OCR adoption rather than test causal hypotheses or develop predictive models. Descriptive statistics are well suited to this objective because they provide a clear representation of participant responses and perceived trends.

**Third**, the inclusion of both expert and user groups enables comparison between different organizational perspectives. Presenting the findings separately for each cohort helps highlight similarities and differences between the two groups without requiring formal between-group statistical testing.

### 3.2 Participant selection and sampling strategy

Participants were selected using a purposive sampling strategy. This approach was adopted because the study required individuals with direct experience in the use, implementation, or management of OCR technology within organizational archiving environments. Given the specialized nature of the target population, probability-based sampling from the general population was not considered appropriate. According to Saunders et al. (2019), purposive sampling is widely used in organizational research when participants are selected on the basis of their relevance to the research objectives rather than statistical representativeness.

To obtain perspectives from different levels of OCR use and implementation, participants were divided into two distinct groups: domain experts and general technology users. This grouping allowed the study to examine OCR adoption from both technical and operational perspectives while maintaining clear separation between the two cohorts. The demographic and organizational characteristics collected for each group are presented in Table 1.

**Table 1:** Demographic and operational profile of participant cohorts.

Demographic attribute	Classification	Experts (*n*=8)	Users (*n*=15)	Total (*N*=23)
**Organizational sector**	Government	7	11	18
	Private	1	4	5
**Organizational size**	< 50 Employees	1	3	4
	50–100 Employees	2	4	6
	101–500 Employees	5	8	13
**OCR adoption duration**	< 1 Year	4	8	12
	1–3 Years	2	5	7
	4–6 Years	2	2	4

Participants were recruited from both public and private sector organizations in Saudi Arabia in order to capture perspectives from organizations of different sizes and varying levels of OCR adoption experience. Including both expert and non-expert participants is consistent with previous research suggesting that perceptions of technology usefulness, ease of use, and organizational impact may vary between technical specialists and everyday system users (Garcia and Claour, 2021).

Sample Size Consideration: the final sample comprised 23 participants, including 8 domain experts and 15 general technology users. Because the study was designed as an exploratory and descriptive investigation using purposive sampling, the sample was intended to provide context-specific perspectives from individuals with direct experience of OCR-related document management rather than to support population-level statistical generalization. Accordingly, the sample size was considered in relation to the exploratory objective and the specialized nature of the target population, rather than as a basis for inferential modeling. The study does not claim that the sample is sufficient for structural equation modeling or other advanced inferential techniques.

### 3.3 Research instrument and operationalization matrix

Data were collected using a structured questionnaire developed specifically for this study, entitled “OCR Adoption and Document Archiving Questionnaire.” The complete questionnaire is provided in Supplementary Appendix 1. It was organized into several sections, each designed to measure a specific aspect of OCR adoption and document archiving. The instrument was developed based on the literature reviewed in Section 2 and aligned with the study objectives and the theoretical framework. Table 2 presents the relationship between research objectives, theoretical constructs, and measurement dimensions used in the study. Table 3 summarizes the structure of the questionnaire, including the distribution of sections and survey items.

- **Section C (Perceived Benefits)**: This section measures the PU construct of the TAM (Davis, 1989). It consists of five items (C1–C5) developed from previous OCR and records management studies (Raj et al., 2023; Benmakhlouf and Chouaou, 2024; Jayoma et al., 2020). These items evaluate participants' perceptions of OCR benefits, including faster document retrieval, improved document accuracy, reduced storage requirements, better information accessibility, and the digitisation of legacy paper records.
- **Section D (Part 1) Organizational Challenges:** This section examines the principal organizational and technical barriers associated with OCR implementation. Seven items (D1–D7), adapted from previous research (Najam and Faizullah, 2023; Alharbi, 2019; Raj et al., 2023; Benmakhlouf and Chouaou, 2024), assess implementation costs, maintenance requirements, the availability of skilled personnel, employee resistance to organizational change, system integration difficulties, risks associated with scanning fragile archival materials, and the adequacy of user training.
- **Section D (Part 2: Integration Simplicity and Perceived Ease of Use–PEOU)**: Based on the TAM (Davis, 1989), this section evaluates participants' overall perception of how easily OCR systems can be integrated into existing organizational workflows. It contains a single four-point categorical item (D8) that measures the perceived simplicity of system integration and day-to-day use.
- **Section E (Perceived Organizational Impact)**: This section assesses participants' perceptions of the organizational outcomes associated with OCR adoption across four operational dimensions (E1–E4) (Raj et al., 2023; Asem et al., 2024). These dimensions include improvements in document retrieval time, document accuracy and integrity, compliance with organizational or regulatory requirements, and overall efficiency in document handling.

**Table 2:** Operationalisation matrix linking study objectives, theoretical constructs, and questionnaire items.

Research objective	Variable/construct	Questionnaire section	Measurement scale
RO1: Contextualize participant and organizational profiles	Demographic Information	Section A	Nominal / Categorical
RO1: Examine current OCR adoption	Adoption Context	Section B	Nominal / Categorical
RO1: Examine perceived OCR usefulness	Perceived Usefulness (PU)	Section C	5-Point Likert scale
RO1: Examine ease of OCR integration	Perceived Ease of Use (PEOU)	Section D (Part 2)	4-Point scale
RO2: Identify implementation challenges	Organizational, Technical, and Operational Challenges	Section D (Part 1)	5-Point Likert scale
RO3: Describe perceived organizational impact	Organizational Impact	Section E	4-Point improvement scale
RO3: Provide supporting qualitative perspectives	Qualitative Commentary	Section F	Open-ended response
RO3: Interpret findings using TAM	TAM as a Conceptual and Interpretive Framework	Sections C, D, and E	Conceptual interpretation; not empirically tested

**Table 3:** Structural composition of the questionnaire.

Section	Purpose	Number of items	Data type
A	Demographic and organizational information	3	Categorical
B	OCR adoption background	2	Categorical
C	Perceived OCR benefits	5	5-Point Likert scale
D	Challenges and integration ease	8	Mixed scale
E	Organizational impact assessment	4	4-Point Improvement scale
F	Open-ended commentary	1	Qualitative response
**Total**	**Complete instrument structure**	**23**	**Mixed descriptive data**

### 3.4 Pilot study and instrument reliability

Prior to the pilot administration, the research instrument was reviewed by three academic experts specializing in Information Systems, Records Management, and Archival Science. The expert review focused on the relevance, clarity, wording, and alignment of the questionnaire items with the study objectives and conceptual framework. Following the expert review, a preliminary pilot study was conducted with a sub-sample of participants (*n* = 6; comprising 3 domain experts and 3 general technology users) to evaluate the operational feasibility, item clarity, and preliminary internal consistency of the instrument. Cronbach's alpha was calculated using 12 five-point Likert-scale items comprising the five Perceived Benefits items (C1–C5) and the seven Perceived Implementation Challenges items (D1–D7). The pilot produced a Cronbach's alpha of α = 0.80, which was treated as preliminary evidence of internal consistency rather than definitive evidence of scale validation given the small pilot sample. Based on the expert feedback and pilot assessment, minor refinements were made where necessary, and the questionnaire was subsequently used in the main study.

### 3.5 Data collection and administration procedure

Data collection was conducted electronically to ensure consistency in questionnaire administration and to facilitate efficient data management. The questionnaire was administered through Google Forms to eligible participants across Saudi Arabian organizations. The complete data collection procedure, from questionnaire distribution to response collection and dataset preparation, is summarized in Figure 2.

**Figure 2:**
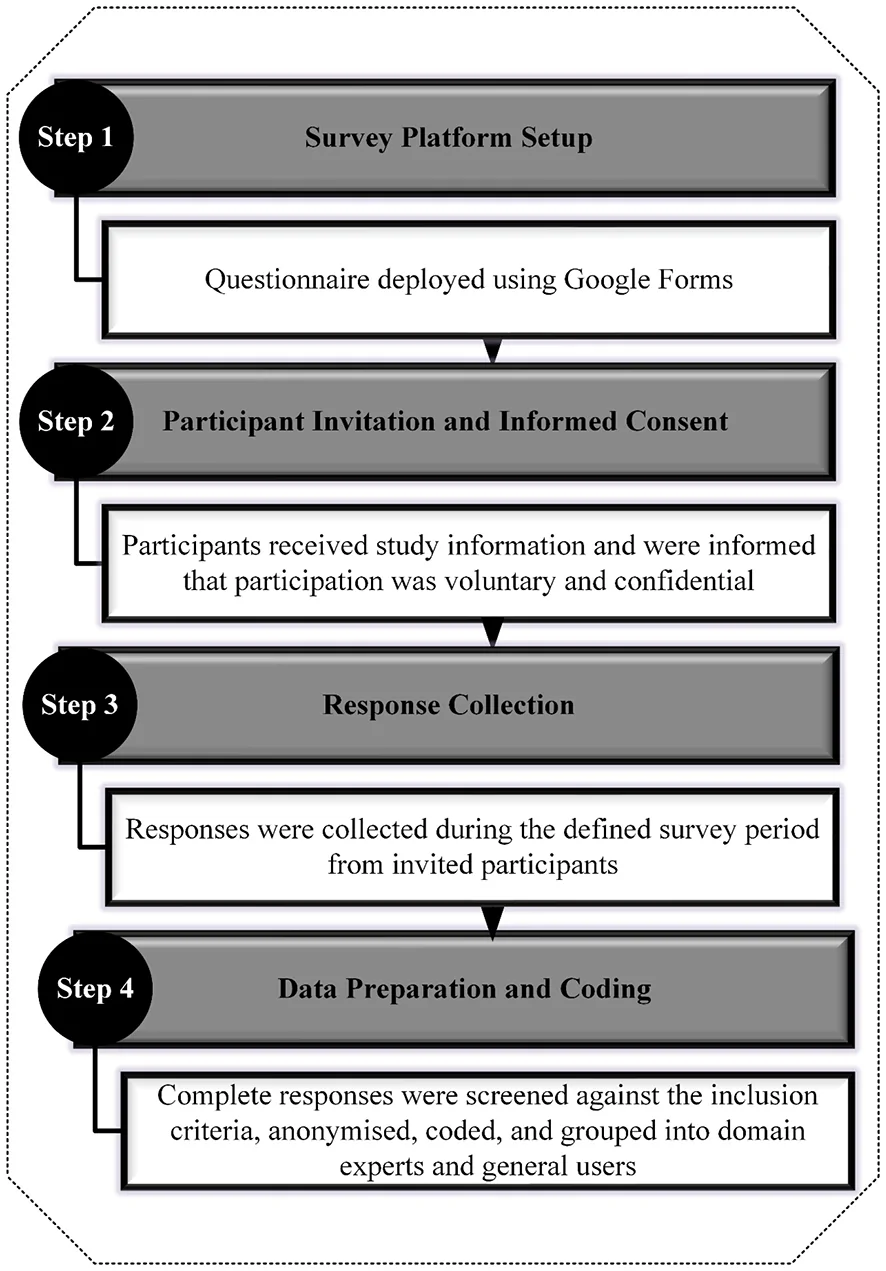
Data collection and administration procedure workflow. The workflow reflects only the documented study procedures, including Google Forms administration, informed consent, response collection, and data preparation and coding.

Participation was entirely voluntary. Before completing the questionnaire, all participants were informed about the purpose of the study, the confidentiality of their responses, and their right to withdraw at any stage without consequence. No personally identifiable information or organizationally sensitive data were collected, ensuring participant anonymity throughout the study.

Participant Inclusion Criteria: eligible participants were required to meet the following criteria: (1) currently employed in a Saudi organization utilizing or evaluating document-archiving workflows; (2) possessing direct experience or operational familiarity with optical character recognition (OCR) tools or paper digitisation practices; and (3) willing to provide informed consent.

Data Collection Protocol: data were collected online using Google Forms over a 7-week period, from 1 October 2025 to 19 November 2025. A direct invitation link, accompanied by an explanatory cover letter and informed consent request, was distributed to a pre-identified pool of 32 prospective participants through professional networks, including LinkedIn and institutional email channels. Of the 32 invited individuals, 23 completed responses were received, yielding a response rate of 71.9%. All received responses satisfied the inclusion criteria and contained no missing data and were therefore retained for the final analysis.

The final dataset was then coded and prepared for analysis using the descriptive statistical procedures described in Section 3.6.

### 3.6 Data analysis procedures

The collected data were analyzed using descriptive statistical techniques. Since the objective of the study was to describe participants' perceptions of OCR adoption rather than test causal relationships or differences between groups, the analysis focused on summarizing response patterns for each participant cohort. Quantitative data from the expert and user groups were analyzed separately, while qualitative responses were reviewed to support the interpretation of the descriptive findings.

Step 1: Preliminary Internal Consistency Assessment

Prior to the main survey, preliminary internal consistency was assessed during the pilot phase using Cronbach's alpha (Nunnally, 1978). The reliability analysis included 12 five-point Likert-scale items: C1–C5 assessing Perceived Benefits and D1–D7 assessing Perceived Implementation Challenges. The pilot yielded α = 0.80. Given that the coefficient was obtained from a six-participant pilot, it is interpreted as preliminary evidence of internal consistency and not as definitive validation of the full instrument.

**Step 2: Frequency and Percentage Distribution**

Frequencies and percentage distributions were calculated for all questionnaire items in Sections C, D, and E to describe the distribution of participants' responses across the rating scales. Percentages were calculated using the following formula:


$$\begin{array}{l} Percentage\;=\;\frac{f}{n}\times 100 \end{array}$$ (1)

where *f* represents the frequency of responses within a particular category and *n* represents the total number of respondents in the corresponding participant group. These percentages were used to describe response patterns for each questionnaire item (Creswell and Creswell, 2017; Saunders et al., 2019).

**Step 3: Weighted Mean Analysis**

Weighted mean (*WM*) scores were calculated to summarize responses for each questionnaire item and facilitate comparisons across the measured variables. The weighted mean was computed using the following formula:


$$\begin{array}{l} WM=\Sigma \left(wf\right)/n \end{array}$$ (2)

where *w* denotes the numerical weight assigned to each response category (1–5 for the five-point Likert scale and 1–4 for the four-point improvement scale), *f* represents the observed frequency of each response category, and *n* denotes the total number of respondents in the corresponding participant group.

The weighted mean scores were interpreted according to the response scales used in the questionnaire.

- **Sections C and D (5-point Likert scale):** Mean scores of 4.00 or above indicate high agreement, scores between 3.00 and 3.99 indicate moderate agreement, and scores below 3.00 indicate low agreement or neutral responses.
- **Section E (4-point improvement scale)**: Mean scores above 2.50 indicate relatively higher perceived organizational improvement, whereas scores of 2.50 or below indicates lower perceived organizational improvement.
- **Sensitivity Consideration:** Given the small size of the expert cohort (n = 8), weighted mean scores were interpreted cautiously. A one-point change in the response of a single expert corresponds to a change of 0.125 in the group mean. Consequently, small differences between item means, particularly within the expert cohort, were not interpreted as evidence of substantive differences unless supported by the broader response distribution and contextual interpretation.

**Step 4: Qualitative Synthesis**

Responses to the open-ended question in Section F were analyzed using open thematic coding. Recurring themes were identified and used to complement the quantitative findings by providing additional context regarding participants' experiences and perceptions of OCR implementation within their organizations.

## 4 Results and empirical findings

This section presents the findings obtained from the cross-sectional survey conducted among two participant groups: domain experts (*n* = 8) and general users (*n* = 15). The results are organized according to the research instrument and are presented using descriptive statistics, including frequencies, percentages, and weighted mean scores. The findings are reported separately for each cohort before a comparative analysis is presented.

### 4.1 Current level of OCR adoption

To address RO1 regarding the current level of OCR technology adoption within the surveyed Saudi organizations, respondents were asked whether their organizations currently use OCR for document archiving or document processing (Item B1) and to indicate the duration of OCR adoption (Item B2).

As shown in Table 4, all 23 respondents (100.0%) confirmed that their organizations currently use OCR technology for document-related activities. Regarding adoption duration, 12 respondents (52.2%) reported that their organizations had adopted OCR for less than 1 year, 7 respondents (30.4%) reported adoption for 1–3 years, and 4 respondents (17.4%) reported adoption for 4–6 years. The participating organizations comprised 18 government-sector organizations (78.3%) and 5 private-sector organizations (21.7%).

**Table 4:** Current OCR adoption status of surveyed organizations (*N* = 23).

Attribute / Question	Classification category	Frequency (f)	Percentage (%)
B1. Current OCR adoption status	Yes	23	100.0%
	No	0	0.0%
B2. OCR adoption duration	< 1 Year	12	52.2%
	1–3 Years	7	30.4%
	4–6 Years	4	17.4%
Organizational sector	Government / Public	18	78.3%
	Private Sector	5	21.7%

### 4.2 Analytical framework

The findings are organized into five analytical sections for each participant group. The analysis begins with demographic and organizational characteristics, followed by participants' perceptions of OCR benefits (Perceived Usefulness), implementation challenges, ease of system integration (Perceived Ease of Use), and perceived organizational impact. The final subsection compares the responses of experts and general users to identify areas of convergence and divergence.

Consistent with the methodology described in Section 3.5, frequencies and percentages are used to describe the distribution of responses, while weighted mean scores summarize the central tendency of each measurement item. These descriptive statistics provide an overview of participants' perceptions without attempting to establish statistical significance or causal relationships.

### 4.3 Expert cohort data (*n* = 8)

#### 4.3.1 Demographic and organizational profiling

This subsection describes the demographic and organizational characteristics of the expert participants to provide context for the subsequent findings. The results are presented in Table 5.

**Table 5:** Demographic and organizational profile of domain experts (*n* = 8).

Variable / Operational attribute	Classification category	Frequency (*f*)	Relative percentage
**Organizational sector**	Government	7	87.5%
	Private	1	12.5%
**Number of employees (Scale)**	< 50 Employees	1	12.5%
	50–100 Employees	2	25.0%
	101–500 Employees	5	62.5%
**OCR adoption duration (Tenure)**	< 1 Year	4	50.0%
	1–3 Years	2	25.0%
	4–6 Years	2	25.0%

Most expert participants were employed in public-sector organizations (87.5%, f = 7), whereas one participant (12.5%) represented the private sector. Regarding organizational size, the largest proportion of respondents (62.5%, f = 5) worked in organizations employing between 101 and 500 staff members.

With respect to OCR experience, half of the experts (50.0%, f = 4) reported less than 1 year of experience using OCR technology. The remaining participants were evenly divided between one to 3 years (25.0%) and 4 to 6 years (25.0%) of experience. Overall, the expert sample included participants from organizations of different sizes and with varying levels of OCR adoption experience.

#### 4.3.2 Perceived benefits (perceived usefulness)

Participants evaluated the perceived benefits of OCR using five statements measured on a five-point Likert scale. The response distributions and weighted mean scores are presented in Table 6.

**Table 6:** Frequency distribution and weighted mean scores for perceived OCR benefits reported by domain experts (*n* = 8).

Benefit item (Section C)	SD	D	N	A	SA	% Agree / SA	Mean score
C1: Increases in document retrieval	0	0	0	4	4	100%	4.50
C2: Enhances accuracy of archived documents	0	1	1	3	3	75.0%	4.00
C3: Reduces physical storage space requirements	0	1	1	3	3	75.0%	4.00
C4: Facilitates easy sharing and access to documents	0	0	1	3	4	87.5%	4.38
C5: Accelerates the archiving of old documents	0	1	0	3	4	87.5%	4.25

SD = Strongly Disagree (1); D = Disagree (2); N = Neutral (3); A = Agree (4); SA = Strongly Agree (5).

Overall, experts reported positive perceptions of OCR benefits, with an overall weighted mean of 4.23 out of 5.00, indicating a high level of agreement across the five benefit items. No participant selected Strongly Disagree for any statement.

Among the individual items, document retrieval efficiency (C1) received the highest rating (M = 4.50), indicating that experts regarded faster document retrieval as the most noticeable benefit of OCR implementation. Information accessibility and document sharing (C4) also received a high rating (M = 4.38).

The remaining benefit items were evaluated positively, with text accuracy (C2) and storage space reduction (C3) each obtaining a weighted mean of 4.00. Although these aspects were viewed favorably, their ratings were slightly lower than those related to retrieval efficiency and information accessibility.

Overall, the results suggest that experts generally perceived OCR as beneficial for organizational document management, particularly in relation to document retrieval and information accessibility.

#### 4.3.3 Perceived challenges and impediments

This subsection presents experts' perceptions of the challenges associated with OCR implementation. Participants evaluated seven potential barriers using a five-point Likert scale. The response distributions and weighted mean scores are presented in Table 7.

**Table 7:** Frequency distribution and weighted mean scores for perceived ocr challenges reported by domain experts (*n* = 8).

Challenge item (Section D, Part 1)	SD	D	N	A	SA	% Agree / SA	Mean score
D1: High initial implementation costs	0	1	2	4	1	62.5%	3.63
D2: Technical issues and maintenance demands	0	1	5	1	1	25.0%	3.13
D3: Lack of skilled personnel	1	0	2	4	1	62.5%	3.50
D4: Resistance to change	2	0	1	3	2	62.5%	3.38
D5: Difficulty in dealing with OCR software	2	0	4	2	0	25.0%	2.75
D6: Sensitivity of old documents to damage	0	1	1	6	0	75.0%	3.63
D7: Lack of training courses on OCR programmes	1	0	2	3	2	62.5%	3.63

Strongly Disagree (1); D = Disagree (2); N = Neutral (3); A = Agree (4); SA = Strongly Agree (5).

Overall, the challenge scale produced a weighted mean of 3.38, indicating a moderate level of agreement with the proposed implementation barriers.

The highest mean score (M = 3.63) was shared by three items: implementation costs (D1), the vulnerability of fragile archival documents (D6), and the limited availability of structured OCR training (D7). Each of these items received agreement from 75.0% of the expert participants, suggesting that they represent commonly perceived challenges.

A relatively high rating was also observed for the shortage of skilled technical personnel (D3) (M = 3.50), indicating that workforce capability remains an important consideration during OCR implementation.

By comparison, software integration difficulties (D5) received the lowest rating (M = 2.75), falling below the neutral midpoint of the scale. This suggests that experts generally regarded software usability as a less prominent challenge than organizational resources, staff development, or the condition of archival materials.

#### 4.3.4 Overall ease of OCR integration

This subsection examines experts' perceptions of the overall ease of integrating OCR systems into existing organizational workflows. Responses were recorded using a four-point rating scale and are summarized in Table 8.

**Table 8:** Expert ratings of the overall ease of integrating OCR into existing systems (*n* = 8).

Scale metric	Difficult (1)	Moderately difficult (2)	Easy (3)	Very easy (4)
Observed frequency (*f*)	1	3	3	1
Relative percentage	12.5%	37.5%	37.5%	12.5%

As shown in Table 8, three experts (37.5%) rated OCR integration as Easy, while another three (37.5%) selected Moderately Difficult. One participant (12.5%) considered the process Very Easy, whereas one (12.5%) rated it as Difficult.

Overall, half of the expert participants (50.0%) viewed OCR integration positively by selecting either Easy or Very Easy, while only one participant reported experiencing difficulty. These findings indicate that, although integration was generally perceived as manageable, experiences varied across organizations. This observation is consistent with previous studies suggesting that the ease of OCR implementation may differ according to organizational infrastructure and the availability of technical support (Gowrishankar and Praveen, 2023).

#### 4.3.5 Organizational impact of OCR technology

This subsection examines experts' perceptions of the organizational impact of OCR implementation across four operational dimensions. Responses were measured using a four-point improvement scale, with weighted mean scores presented in Table 9.

**Table 9:** Expert ratings of the organizational impact of OCR technology (*n* = 8).

Impact dimension (Section E)	Slightly (1)	Moderately (2)	Highly (3)	Extremely (4)	Mean score
E1: Document retrieval time	0.0% (0)	37.5% (3)	37.5% (3)	25.0% (2)	2.88
E2: Document accuracy & integrity	25.0% (2)	25.0% (2)	37.5% (3)	12.5% (1)	2.38
E3: Compliance with regulatory needs	0.0% (0)	62.5% (5)	25.0% (2)	12.5% (1)	2.50
E4: Overall document handling efficiency	12.5% (1)	50.0% (4)	12.5% (1)	25.0% (2)	2.50

Among the four dimensions, document retrieval time (E1) received the highest weighted mean (M = 2.88). Approximately 62.5% of the experts reported either High Improvement or Extreme Improvement, indicating that faster document retrieval was the most consistently perceived organizational benefit.

Regulatory compliance (E3) and overall document handling efficiency (E4) each recorded a weighted mean of 2.50, reflecting moderate perceived improvements following OCR implementation.

The lowest mean score was observed for text accuracy and data integrity (E2) (M = 2.38). In addition, one-quarter of the participants reported only slight improvements for this item. These responses suggest that experts perceived the effect of OCR on document accuracy less consistently than its effect on document retrieval and workflow efficiency.

#### 4.3.6 Qualitative commentary

This subsection summarizes the qualitative comments provided by participants through the optional open-ended question. These responses are presented to complement the quantitative findings by offering additional contextual observations.

Among the eight expert participants, six (75.0%) did not provide additional comments.

One expert explained that although their organization operates largely through automated digital systems, incoming paper-based correspondence continues to require manual processing before OCR can be applied. This observation highlights the continuing role of OCR in supporting the digitisation of external documents.

Another expert noted that their organization considers digital technologies an important component of library services and information governance, reflecting the broader organizational emphasis on digital transformation.

### 4.4 General user cohort data (*n* = 15)

#### 4.4.1 Perceived benefits (perceived usefulness)

This subsection presents general users' perceptions of the benefits of OCR technology (Section C). Responses were measured using the standard five-point Likert scale, ranging from 1 = Strongly Disagree to 5 = Strongly Agree, consistent with the questionnaire specifications presented in Supplementary Appendix 1 and Table 10.

**Table 10:** Frequency distribution and weighted mean scores for perceived OCR benefits reported by general users (*n* = 15).

Benefit item (Section C)	SD	D	N	A	SA	% Agree / SA	Mean score
C1: Increased document retrieval efficiency	0	0	0	7	8	100%	4.53
C2: Enhanced accuracy of archived documents	0	1	1	7	6	86.7%	4.27
C3: Reduces physical storage requirements	0	2	1	7	5	80.0%	4.00
C4: Facilitates easy sharing and access	0	0	1	6	8	93.3%	4.47
C5: Accelerates archiving of old documents	0	0	1	7	7	93.3%	4.40

The overall weighted mean for the benefits scale was 4.33 out of 5.00, indicating generally positive perceptions of OCR among general users. No participant selected Strongly Disagree for any of the five benefit items.

The highest-rated benefit was document retrieval efficiency (C1) (M = 4.53), with all participants selecting either Agree or Strongly Agree. Information accessibility and document sharing (C4) also received a high rating (M = 4.47).

The lowest weighted mean was recorded for physical storage space reduction (C3) (M = 4.00). Although participants generally agreed that OCR contributes to reducing storage requirements, this benefit received a lower rating than those related to retrieval efficiency and information accessibility.

#### 4.4.2 Perceived challenges and impediments

This subsection presents general users' perceptions of the challenges associated with OCR implementation. Responses were measured using a five-point Likert scale, and the findings are presented in Table 11.

**Table 11:** Frequency distribution and weighted mean scores for perceived OCR challenges reported by general users (*n* = 15).

Challenge item (Section D, Part 1)	SD	D	N	A	SA	% Agree / SA	Mean score
D1: High initial implementation costs	0	1	4	8	2	66.7%	3.73
D2: Technical issues and maintenance demands	1	0	11	2	1	20.0%	3.13
D3: Lack of skilled personnel	1	0	5	8	1	60.0%	3.53
D4: Resistance to change	4	0	3	5	3	53.3%	3.20
D5: Difficulty in dealing with OCR software	4	0	8	3	0	20.0%	2.67
D6: Sensitivity of old documents to damage	0	1	3	11	0	73.3%	3.67
D7: Lack of training courses	1	0	5	4	5	60.0%	3.80

SD = Strongly Disagree (1); D = Disagree (2); N = Neutral (3); A = Agree (4); SA = Strongly Agree (5).

The overall weighted mean for the challenge scale was 3.39, indicating a moderate level of agreement with the proposed implementation barriers.

Among the evaluated items, the limited availability of OCR training programmes (D7) received the highest weighted mean (M = 3.80). Implementation costs (D1) (M = 3.73) and the condition of fragile archival documents (D6) (M = 3.67) also received relatively high ratings.

The lowest weighted mean was recorded for software integration difficulties (D5) (M = 2.67), suggesting that users generally regarded software usability as a less prominent implementation challenge.

Responses to employee resistance to change (D4) were more varied. While just over half of the participants (53.3%) agreed that resistance to change represented a challenge, 26.7% selected Strongly Disagree, indicating that experiences differed across organizations.

#### 4.4.3 Overall ease of OCR use (perceived ease of use)

This subsection presents users' perceptions of the overall ease of using OCR systems. Responses were collected using a four-point rating scale and are summarized in Table 12.

**Table 12:** General user ratings of the overall ease of OCR use (*n* = 15).

Scale metric	Difficult (1)	Moderately difficult (2)	Easy (3)	Very easy (4)
Observed frequency (*f*)	3	5	5	2
Relative percentage	20.0%	33.3%	33.3%	13.3%

The overall weighted mean was 2.40, indicating that participants generally perceived OCR integration as moderately easy.

Almost half of the participants (46.7%, f = 7) rated OCR use as Easy or Very Easy, whereas three participants (20.0%) reported that using the system was Difficult. These findings suggest that, although many users considered OCR relatively easy to use, some continued to experience usability challenges during routine operations.

#### 4.4.4 Organizational impact of OCR technology

This subsection presents users' perceptions of the organizational impact of OCR implementation across four operational dimensions. The results are summarized in Table 13.

**Table 13:** General user ratings of the organizational impact of OCR technology (*n* = 15).

Impact dimension (Section E)	Slightly (1)	Moderately (2)	Highly (3)	Extremely (4)	Mean score
E1: Document retrieval time	0.0% (0)	40.0% (6)	26.7% (4)	33.3% (5)	2.93
E2: Document accuracy & integrity	20.0% (3)	26.7% (4)	40.0% (6)	13.3% (2)	2.47
E3: Compliance with regulatory needs	0.0% (0)	60.0% (9)	26.7% (4)	13.3% (2)	2.53
E4: Overall document handling efficiency	6.7% (1)	53.3% (8)	6.7% (1)	33.3% (5)	2.67

The overall weighted mean for organizational impact was 2.65 out of 4.00.

Among the four dimensions, document retrieval time (E1) received the highest weighted mean (M = 2.93), with 60.0% of participants reporting either High Improvement or Extreme Improvement.

Overall document handling efficiency (E4) recorded the second-highest mean (M = 2.67), followed by regulatory compliance (E3) (M = 2.53).

The lowest rating was observed for text accuracy and data integrity (E2) (M = 2.47). Similar to the expert cohort, users perceived improvements in document retrieval more consistently than improvements in document accuracy.

### 4.5 Cross-group comparative analysis

This subsection compares the responses of domain experts and general users to identify similarities and differences in their perceptions of OCR implementation. A summary of the comparative findings is presented in Table 14.

- Perceived Usefulness (PU): Both participant groups reported positive perceptions of the benefits of OCR. The overall weighted mean was 4.23/5.00 among domain experts and 4.33/5.00 among general users. In both cohorts, document retrieval efficiency (C1) and information accessibility (C4) received the highest item-level ratings, while physical storage space reduction (C3) received the lowest item-level rating within the benefit set.
- **Perceived Challenges:** Similar response patterns were also observed for several implementation challenges. Software integration difficulties (D5) received relatively low ratings among both experts (M = 2.75) and users (M = 2.67), while technical maintenance requirements (D2) yielded a weighted mean of 3.13 for both participant groups after rounding to two decimal places, although the underlying response distributions differed between the expert and general-user cohorts. These findings suggest that software usability was not viewed as the most significant implementation challenge by either group.
- **Training Requirements**: A noticeable difference was observed for OCR training (D7). General users reported a slightly higher weighted mean (M = 3.80) than experts (M = 3.63), indicating that users perceived training needs more strongly than the expert cohort.
- **Perceived Ease of Use (PEOU)**: The overall ratings for ease of integration were similar between experts (M = 2.50) and general users (M = 2.40). Both means were close to the midpoint of the scale, suggesting broadly comparable perceptions of OCR integration across the two participant groups.
- **Organizational Impact**: General users reported a slightly higher overall organizational impact score (M = 2.65) than experts (M = 2.57). Although the difference was small, it indicates that users tended to perceive somewhat greater improvements following OCR implementation than the expert participants.

**Table 14:** Comparison of expert and general user perceptions.

Analytical dimension	Expert metric (From baseline tables)	User metric (From baseline tables)	Scale equivalence / Trend	Primary cross-level operational dynamic
Perceived usefulness (PU)	4.23 / 5.00 (Table 6)	4.33 / 5.00 (Table 10)	Comparable five-point scale	Both groups reported positive perceived usefulness; C1 received the highest item-level rating in each group.
Perceived challenges (Usability)	2.75 / 5.00 (Table 7)	2.67 / 5.00 (Table 11)	Descriptive low-to-moderate barrier pattern	D5 received a relatively low mean in both groups (2.75 among experts; 2.67 among users).
Perceived challenges (Training)	3.63 / 5.00 (Table 7)	3.80 / 5.00 (Table 11)	Descriptive difference	General users reported a slightly higher mean for training requirements (3.80) than domain experts (3.63).
Perceived ease of use (PEOU)	2.50 / 4.00 (Table 8)	2.40 / 4.00 (Table 12)	Similar descriptive means	The overall ease-of-integration means were 2.50 among experts and 2.40 among general users.
Organizational impact assessment	2.57 / 4.00 (Table 9)	2.65 / 4.00 (Table 13)	Slight descriptive difference	General users reported an overall mean of 2.65 compared with 2.57 among domain experts.

## 5 Discussion

This section interprets the empirical findings in relation to the study objectives and the theoretical perspective provided by the TAM. The discussion integrates the quantitative findings obtained from both participant groups' domain experts (*n* = 8) and general users (*n* = 15) with previous research on OCR technology, digital transformation, and records management. Rather than examining the results in isolation, the section explores how participants' perceptions of OCR adoption reflect both technological capabilities and organizational conditions within Saudi Arabian institutions.

### 5.1 Perceived usefulness (PU)

The findings indicate a consistently positive perception of the practical value of OCR technology, supporting the PU construct proposed by Davis (1989). Across both participant groups, document retrieval efficiency emerged as the most highly valued benefit of OCR implementation. Complete agreement on item C1 demonstrates that participants regarded rapid access to archived documents as the technology's principal contribution. This result reflects the operational reality of modern organizations, where replacing manual paper-based retrieval with searchable digital archives can substantially reduce the time required to locate information.

In contrast, the perceived benefit associated with reducing physical storage requirements (item C3) received a comparatively lower rating than the benefits related to document retrieval and information accessibility. This finding indicates that participants placed greater emphasis on accessibility and workflow efficiency than on the reduction of physical storage requirements. However, the present questionnaire did not directly examine the organizational reasons underlying the lower rating for physical storage reduction. Therefore, this result should be interpreted as a descriptive perception rather than as evidence of a specific underlying cause.

These findings are consistent with previous studies that identify faster document retrieval and improved information accessibility as the primary organizational benefits of OCR adoption (Benmakhlouf and Chouaou, 2024; Hegghammer, 2022; Jayoma et al., 2020). They also support the broader argument that user evaluations of OCR increasingly depend on its contribution to operational performance rather than on its basic digitisation capabilities.

### 5.2 Organizational challenges

Although participants generally recognized the usefulness of OCR technology, the findings also reveal several organizational and technical barriers that may influence successful implementation. Among all identified challenges, insufficient training opportunities (item D7) emerged as one of the most prominent concerns across both participant groups. This suggests that acquiring OCR software alone is insufficient to ensure effective adoption. Organizations must also provide appropriate training to enable employees to use the technology efficiently and confidently.

The findings further indicate that limited training is closely associated with concerns regarding the handling of fragile historical documents (item D6). Participants expressed reservations about digitizing valuable archival materials without adequate knowledge of appropriate scanning procedures and document preservation techniques. These concerns highlight the importance of combining technological implementation with organizational capacity building.

Interestingly, these findings reveal a contrast within the Saudi organizational environment. While many institutions have made substantial progress in digital transformation and employees generally possess adequate digital literacy, specialized competencies required for professional OCR implementation remain comparatively limited. This suggests that successful OCR adoption depends not only on technological readiness but also on continuous workforce development and organizational support.

The results therefore reinforce previous studies that identify employee preparedness, organizational training, and change management as important determinants of successful digital transformation initiatives (Alharbi, 2019; Asem et al., 2024; Abudaqqa, 2025).

### 5.3 Perceived ease of use (PEOU)

The findings related to overall ease of use provide additional insight into participants' perceptions of OCR implementation within organizational environments. The results indicate that approximately half of the respondents considered integrating OCR into existing systems to be relatively straightforward, suggesting that the technology is generally viewed as manageable rather than inherently difficult to implement.

However, the remaining responses indicate that integration challenges continue to exist in certain organizational settings. Previous OCR research has reported that Arabic-language documents may present additional processing difficulties associated with cursive writing, variations in character forms, ligatures, diacritical marks, and degraded archival materials (Najam and Faizullah, 2023), (Kasem et al., 2026). These findings provide contextual background for interpreting OCR integration challenges in Arabic-language archival environments. However, these Arabic-script characteristics were not directly measured in the present survey and should therefore not be treated as empirical findings of this study.

Taken together, the survey findings and the relevant literature suggest that the perceived ease of use of OCR may be influenced not only by software usability but also by document complexity and language-specific characteristics. Consequently, although current OCR platforms generally provide user-friendly interfaces, successful implementation still depends on document quality, preprocessing procedures, and organizational support mechanisms.

This interpretation is consistent with previous OCR research, which suggests that ease of implementation is shaped by both technological capability and operational context rather than software design alone (Gowrishankar and Praveen, 2023; Najam and Faizullah, 2023; Kasem et al., 2026).

### 5.4 Organizational impact

The results indicate that OCR technology contributes positively to organizational performance, particularly in areas related to document retrieval and information accessibility. Among all organizational impact indicators, reducing document retrieval time (item E1) received the highest evaluation from both participant groups, demonstrating that respondents perceive immediate operational benefits following OCR implementation.

These findings are consistent with previous studies reporting that OCR enhances workflow efficiency by enabling rapid access to archived information and reducing dependence on manual document retrieval procedures (Benmakhlouf and Chouaou, 2024; Hegghammer, 2022; Jayoma et al., 2020). Within the Saudi Arabian context, these operational improvements also support the broader objectives of digital transformation initiatives associated with Vision 2030 by facilitating more efficient document management and electronic service delivery.

Nevertheless, participants assigned comparatively lower ratings to improvements in document accuracy and information integrity. This suggests that although OCR effectively accelerates access to information, recognition errors remain a concern, particularly when processing Arabic documents or low-quality archival materials. Consequently, OCR should be viewed as an enabling technology that supports organizational efficiency rather than as a complete substitute for document verification and quality assurance procedures.

The findings therefore suggest that the greatest organizational value of OCR lies in improving workflow efficiency and information accessibility, while achieving broader governance objectives will require closer integration with document management systems, metadata standards, and institutional information governance frameworks.

### 5.5 Synthesis and strategic recommendations

Combining the quantitative findings with participants' qualitative comments provides a comprehensive picture of OCR adoption within Saudi Arabian organizations. Overall, respondents recognized the operational value of OCR while simultaneously identifying several organizational conditions that influence successful implementation.

#### 5.5.1 TAM as an interpretive framework

The findings also illustrate how the Technology Acceptance Model (TAM) can serve as a useful interpretive framework for organizing participants' perceptions of OCR adoption. This interpretation supports RO3 while recognizing that TAM is not empirically tested in the present study.

Based on these findings, three practical recommendations can be proposed.

**First**, organizations should establish structured training programmes that extend beyond basic software operation to include document preparation, quality assurance, and best practices for processing Arabic-language archival materials. Continuous training would improve user confidence and reduce implementation-related challenges.

**Second**, software developers should continue improving Arabic-language OCR capabilities by enhancing support for cursive text, historical documents, and complex page layouts. User-friendly interfaces and localization features would further improve the overall user experience, particularly for non-technical employees.

**Third**, OCR implementation should be integrated within broader organizational information management strategies rather than treated as an isolated digitisation initiative. Linking OCR systems with document management platforms, metadata generation, and compliance monitoring tools would maximize their contribution to institutional efficiency and long-term information governance.

Collectively, these recommendations support a more sustainable approach to OCR adoption by addressing both technological and organizational dimensions identified throughout this study.

## 6 Conclusion and future directions

### 6.1 Conclusion

This exploratory study examined the current adoption and perceived organizational implications of Optical Character Recognition (OCR) technology within Saudi Arabian document-archiving contexts. The findings are based on responses from domain experts (*n* = 8) and general technology users (*n* = 15) and are intended to provide descriptive, context-specific evidence rather than population-level estimates or causal explanations.

The results indicate that all surveyed participants reported that their organizations currently use OCR for document-related activities. Across both participant groups, the strongest perceived benefit was faster document retrieval, reflecting the high perceived usefulness of OCR for improving access to archived information. At the same time, respondents identified implementation concerns related particularly to training, implementation costs, and the handling of fragile archival materials. Perceptions of ease of integration were more mixed, indicating that OCR adoption may require appropriate technical preparation and organizational support.

The findings also illustrate the usefulness of the Technology Acceptance Model (TAM) as an interpretive framework for organizing perceptions of OCR usefulness and ease of integration. However, the present study did not test the causal relationships or predictive validity of TAM. The findings should therefore be interpreted as exploratory evidence of participants' perceptions within the surveyed organizations rather than as empirical validation of the TAM model.

Overall, the study provides a descriptive account of OCR adoption and perceived organizational impact within the participating Saudi organizations. It highlights the practical value of OCR while also demonstrating the importance of training, implementation planning, document conditions, and organizational support. Future research using larger and more diverse samples, objective OCR performance measures, and inferential modeling could provide broader and more robust evidence regarding OCR adoption and its organizational outcomes.

### 6.2 Future directions

While the study provides some descriptive evidence on OCR's adoption by Saudi Arabian organizations, it also presents numerous opportunities for future research. Initially, longitudinal studies would allow researchers to investigate the changes in both user views and organization outcomes over time as a result of OCR adoption. Such studies could shed additional light on technology acceptance and organizational learning in other stages of digital transformation. Second, future studies need to recruit a larger and more heterogeneous sample comprising a wider range of government and non-state sectors. A wider range of participant samples would be beneficial for generalizability of findings and comparative purposes across different organizational contexts. Third, the effectiveness of new Arabic OCR models under standard archival conditions is requiring further technical research. The handwritten text recognition, the historical manuscripts, degraded documents and the complex document layouts that still pose major recognition challenges should be taken into special account. Finally, new research work may build upon the proposed descriptive framework by using inferential statistical methods including Structural Equation Modeling (SEM) or Partial Least Squares Structural Equation Modeling (PLS-SEM). These methodologies would enable researchers to analyse the causal linkages between the constructs of the Technology Acceptance Model and determine if organizational factors affect OCR adoption behavior.

## 7 Limitations

The results should be interpreted in light of several limitations. First, the study used a relatively small purposive sample of 23 participants who had prior experience or operational familiarity with OCR in Saudi Arabian organizations. This selection approach was appropriate for the exploratory objective but may introduce selection bias because individuals with relevant OCR experience may have perceptions that differ from those of employees or organizations with limited or no prior exposure to the technology. Second, the small sample size limits the statistical generalizability of the findings to the wider population of Saudi Arabian organizations, and the results should therefore be understood primarily as context-specific exploratory evidence. Third, the study examined participants' perceptions of organizational outcomes rather than objective measures such as OCR recognition accuracy, processing time, cost savings, or archival performance. Finally, the cross-sectional design captures perceptions at one point in time and does not permit conclusions about changes in perceptions or organizational outcomes over time.

## Data Availability

The original contributions presented in the study are included in the article/Supplementary material, further inquiries can be directed to the corresponding author.
