# Supplementary material for: Exploring perceptions of OCR technology adoption for paper document archiving in Saudi Arabia: an exploratory study
Source: Front Big Data. 2026 Oct 29;9:1916400. doi: 10.3389/fdata.2026.1916400 (PMC13754831; doi:10.3389/fdata.2026.1916400)
Supplement: Supplementary file 1 [file Data_Sheet_1.docx]

**Appendix 1: OCR Adoption and Document Archiving Questionnaire**

**Research Title**

**Exploring the Adoption and Organizational Impact of OCR Technology for Paper Document Archiving in Saudi Arabia: A TAM-Based Assessment**

Dear Participant,

You are invited to participate in this academic research study examining the adoption and use of Optical Character Recognition (OCR) technology in document archiving environments within organisations in the Kingdom of Saudi Arabia.

Your responses will contribute to understanding the perceived benefits, implementation challenges, ease of integration, and organisational impact of OCR systems. Participation is entirely voluntary, and all responses will remain confidential and anonymous. The questionnaire is intended solely for academic research purposes.

Estimated completion time: **7–10 minutes**

**Section A: Organisational and Demographic Information**

Please select the option that best describes your organisation and role.

**A1. Organisational Sector**

- ☐ Government / Public Sector
- ☐ Private Sector

**A2. Approximate Number of Employees in Your Organisation**

- ☐ Less than 50 employees
- ☐ 50–100 employees
- ☐ 101–500 employees
- ☐ More than 500 employees

**A3. Participant Category**

- ☐ Domain Expert (OCR implementation, document management, records management, IT administration)
- ☐ General User (Uses OCR systems without implementation responsibilities)

**Section B: OCR Adoption Background**

**B1. Does your organisation currently use OCR technology for document archiving or document processing?**

- ☐ Yes
- ☐ No

**B2. How long has OCR technology been used in your organisation?**

- ☐ Less than 1 year
- ☐ 1–3 years
- ☐ 4–6 years
- ☐ More than 6 years

**Section C: Perceived Benefits of OCR Technology**

**Instructions**

Please indicate your level of agreement with the following statements regarding the benefits of OCR technology.

| **Scale** | **Meaning** |
| --- | --- |
| 1 | Strongly Disagree |
| 2 | Disagree |
| 3 | Neutral |
| 4 | Agree |
| 5 | Strongly Agree |

| **No.** | **Statement** | **1** | **2** | **3** | **4** | **5** |
| --- | --- | --- | --- | --- | --- | --- |
| C1 | OCR technology improves document retrieval efficiency. | ☐ | ☐ | ☐ | ☐ | ☐ |
| C2 | OCR enhances the accuracy and integrity of archived documents. | ☐ | ☐ | ☐ | ☐ | ☐ |
| C3 | OCR reduces physical storage requirements. | ☐ | ☐ | ☐ | ☐ | ☐ |
| C4 | OCR facilitates easier document sharing and accessibility. | ☐ | ☐ | ☐ | ☐ | ☐ |
| C5 | OCR accelerates the digitisation and archiving of legacy documents. | ☐ | ☐ | ☐ | ☐ | ☐ |

**Section D: Challenges and Ease of Integration**

**Part 1: Challenges Associated with OCR Adoption**

**Instructions**

Please indicate your level of agreement with the following challenges related to OCR implementation and use.

| **Scale** | **Meaning** |
| --- | --- |
| 1 | Strongly Disagree |
| 2 | Disagree |
| 3 | Neutral |
| 4 | Agree |
| 5 | Strongly Agree |

| **No.** | **Statement** | **1** | **2** | **3** | **4** | **5** |
| --- | --- | --- | --- | --- | --- | --- |
| D1 | High implementation costs represent a significant challenge. | ☐ | ☐ | ☐ | ☐ | ☐ |
| D2 | OCR systems require substantial technical maintenance and support. | ☐ | ☐ | ☐ | ☐ | ☐ |
| D3 | Lack of skilled personnel limits effective OCR implementation. | ☐ | ☐ | ☐ | ☐ | ☐ |
| D4 | Employees may resist organisational transition toward OCR systems. | ☐ | ☐ | ☐ | ☐ | ☐ |
| D5 | OCR software can be difficult to integrate into existing workflows. | ☐ | ☐ | ☐ | ☐ | ☐ |
| D6 | Historical or fragile documents may be damaged during scanning processes. | ☐ | ☐ | ☐ | ☐ | ☐ |
| D7 | Insufficient training negatively affects OCR adoption effectiveness. | ☐ | ☐ | ☐ | ☐ | ☐ |

**Part 2: Ease of OCR Integration**

**D8. How would you rate the overall ease of integrating OCR technology into existing organisational systems?**

- ☐ Difficult
- ☐ Moderately Difficult
- ☐ Easy
- ☐ Very Easy

**Section E: Perceived Organisational Impact**

**Instructions**

Please evaluate the extent to which OCR technology has improved the following organisational dimensions.

| **Scale** | **Meaning** |
| --- | --- |
| 1 | Slightly Improved |
| 2 | Moderately Improved |
| 3 | Highly Improved |
| 4 | Extremely Improved |

| **No.** | **Statement** | **1** | **2** | **3** | **4** |
| --- | --- | --- | --- | --- | --- |
| E1 | Improvement in document retrieval time | ☐ | ☐ | ☐ | ☐ |
| E2 | Improvement in document accuracy and integrity | ☐ | ☐ | ☐ | ☐ |
| E3 | Improvement in compliance with organisational or regulatory requirements | ☐ | ☐ | ☐ | ☐ |
| E4 | Improvement in overall document handling efficiency | ☐ | ☐ | ☐ | ☐ |

**Section F: Open-Ended Feedback**

**F1. Please provide any additional comments, experiences, or recommendations regarding the use of OCR technology within your organisation.**

....................................................................................

....................................................................................

....................................................................................

....................................................................................

**Informed Consent Statement**

By completing this questionnaire, you confirm that:

- Your participation is voluntary.
- You understand the purpose of the study.
- You may withdraw at any time before submission.
- Your responses will remain anonymous and confidential.
- The collected data will be used exclusively for academic research purposes.

**Thank You**

Thank you for your valuable participation and contribution to this research study.
